# “They just left me”: people seeking asylum, mental and physical health, and structural violence in the UK’s institutional accommodation

**DOI:** 10.3389/fpubh.2025.1454548

**Published:** 2025-03-12

**Authors:** Jenny Phillimore, Lin Fu, Lucy Jones, Laurence Lessard-Phillips, Briony Tatem

**Affiliations:** ^1^Department of Social Policy, Sociology and Criminology, University of Birmingham, Birmingham, United Kingdom; ^2^Institute of Applied Health Research, University of Birmingham, Birmingham, United Kingdom; ^3^Doctors of the World UK, London, United Kingdom

**Keywords:** institutional accommodation, people seeking asylum, structural violence, mental health, access to healthcare

## Abstract

This paper presents the findings of a novel post-hoc analysis of data collected by Doctors of the World UK (DOTW UK) between 2020 and 2022 from people seeking asylum housed in repurposed hotels and barracks in England. We analyzed qualitative and quantitative data on individuals’ mental and physical health using a structural violence analytical framework. Institutional housing and associated poor living conditions were reported to contribute deteriorations in physical and mental health which collectively undermined wellbeing. Inactions around urgent and life-threatening health conditions were seen as placing individuals’ lives and long-term health at risk. Provision of housing does not currently meet people’s needs and actively undermines their mental and physical health. We conclude that approaches to accommodation provision need to be adapted to ensure individuals’ right to health is realized. We outline recommendations for policy and future research.

## 1 Introduction

Global displacement reached an all-time high in 2022 with over 108 million people living out of place having been forced to migrate in search of safety (1). Most displaced people move within their country or to those nearby, often accommodated in camps or in urban areas. By 2022 around 5.4 million people were seeking asylum, with Europe, one of the largest destination regions, receiving nearly 50% of these applications (1) and the UK 81,130 applications, the highest number since 2002 (2). With asylum numbers increasing, states have experienced pressure identifying housing for new arrivals. As a result, housing which used to be framed as temporary is being used for longer periods. The UK has contracted out asylum housing to private subcontractors since 2012. During the pandemic there was a huge shift from the use of subcontracted private sector, community-based, housing to the use of an institutional housing model. Hotels are now the main source of accommodation for individuals and families, with more than 47,000 individuals residing in contingency accommodation across the UK in December 2023 (3). Other forms of institutional housing such as ex-army barracks and a barge have also been utilized. The use of facilities not designed for long-term residence in areas lacking capacity to support vulnerable people seeking asylum has been widely criticized. Criticisms have largely focused on costs and concerns about “security” driven by the former Government discourse seeking to portray people, and especially those arriving via small boats seeking asylum, as so-called illegal immigrants taking advantage of the British taxpayer (4). Little attention has been paid to concerns expressed by NGOs and human rights organizations about the unsuitability of hotels, and barracks, for the health and wellbeing of people seeking asylum (5). We offer novel insight into the health and wellbeing of those residing in institutional housing applying a structural violence analytical framework following a post-hoc analysis of qualitative and quantitative data collected by clinicians to examine the ways in which housing provision may influence their health and access to healthcare and cause harm.

Access to adequate housing is a human right (6) that extends beyond shelter to offer a place “which protects privacy, contributes to physical and psychological wellbeing and supports the development and social integration of its inhabitants” [(7), p. 413]. Housing has also been identified as a means and marker of refugee integration by the UK Government, recognizing the importance of appropriate and stable accommodation to the long-term wellbeing of refugees (8). Further, housing has long been established as a key social determinant of health with an extensive body of research linking housing to health and wellbeing (9). Silove et al. (10) warned two decades ago that the use of non-standard housing such as military barracks, detention centers, and other institutional-style accommodation of people seeking asylum would have significant effects on mental and physical health (11). Yet the former-UK Government set out plans to shift housing of all newly arrived people seeking asylum to institutional housing outlining intentions to make this housing as basic as possible (12) and under the new UK Government hotels and barracks continue to be used for the main forms of housing. There is an urgent need to understand the ways in which such accommodation is linked to access to healthcare and health and wellbeing and how this situation might be researched in the future.

In this paper, we ask the following question: in what ways does residing in institutional housing influence the health and access to healthcare of people seeking asylum? We use a structural violence framework to make the case that such accommodation is linked to the generation of health and wellbeing harms for vulnerable individuals. The paper begins with a discussion of the policies that have led to the reliance on hotel accommodation, a review of the state of knowledge on health and institutional housing and an outline of our structural violence framework. We then move to outline our methods including a discussion of the data and analytical techniques before setting out our findings. We end the paper with a discussion of our contribution to knowledge, the importance of the right to health, key limitations of the study and some suggestions for future research.

## 2 Housing people seeking asylum

In 2000, the UK introduced a new support system shifting people seeking asylum away from support within the welfare state to removing them from mainstream systems and introducing a National Asylum Seeker Support Service (NASS). The rationale behind this was to ‘spread the load’ from London and the South-East where most of the costs of asylum support fell (13). Two asylum accommodation models were implemented. The first was a community-based approach in which individuals are dispersed to locations with housing availability to live independently within standard housing. Until the ‘refugee crisis’ of 2015/16 Germany, Austria, Sweden, and the UK predominantly utilized this model (14). In the UK this housing was mostly provided by local authorities until 2012 using unpopular social housing in areas of housing market decline (15). The model shifted from the public to the private sector in 2012 as the Government contracted with three private companies who themselves sub-contracted private sector accommodation in the lowest cost areas of the UK in a bid to maximize profits. The shift to the private sector was framed as a drive for fiscal savings within the context of an increasingly neo-liberal agenda enabled through the discourse of austerity (15). Darling (15) argued this shift came at the expense of human dignity.

The second model implemented in Europe involved the use of institutional housing. Several EU countries have purpose-built accommodation or reception centers wherein people seeking asylum remain while their claims are processed. Often the processing times for asylum claims, and consequently length of stay, is highly regulated (16). Over time, and with an increase in arrivals, such as of Syrians in 2015/16 and Ukrainians in 2022, other initially temporary solutions have been identified. These include the use of tents, schools, containers, boats, the repurposing of abandoned buildings (17) and homestay (18). Continued arrivals coupled with unprecedented delays in processing claims, and a shortage of move-on housing have led to a situation where these once temporary situations are being utilized for lengthy periods.

The UK has long utilized institutional housing, including purpose-built asylum hostels and occasionally hotels, for small numbers of new arrivals. Stays were intended to last 35 days or less. Hotels vary enormously from hostel-like accommodation to those viewed as more comfortable (19). Since 2019, there has been a vast increase in the number of individuals living in initial/contingency accommodation (3, 20). Most recently a policy of increasing occupancy of hotel rooms by doubling, tripling or quadrupling the number of beds has reduced the number of hotels in use from 400 to 320 (3). Individuals are now accommodated in institutional housing for lengthy periods with most resident for at least 6 months, but some housed in such accommodation for over 2 years (21). At the same time community dispersal has declined. High profile incidents, such as the death of a person seeking asylum from diphtheria at the chronically overcrowded Manston Reception Center (20), and the fatal shooting of a psychologically distressed person seeking asylum accommodated at a hotel in Glasgow (22), point to the health implications for those living in such accommodation. Yet, the main Government concern regards costs said to be more than £1.3 billion a year at April 2024 (3), paid to sub-contractors to contract hotels often in areas that lack infrastructure around the support of people seeking asylum. Almost no attention is paid to health and wellbeing while living long-term in institutional housing. Further, although the UK Government is legally obliged to ensure access to healthcare (23) it is unclear the extent to which individuals can reach the services they need. Certainly, the individual shot in Glasgow after attacking fellow residents had made repeated attempts to seek help with his psychological condition (22, 74).

The previous government was progressing plans to develop further institutional accommodation as part of their immigration reforms and they intended that individuals seeking asylum would remain in semi-detention until they could be deported. This move was widely condemned globally and nationally and it is not clear what actions the new UK Government will take. The contract for the mooring of a barge off the coast in the rural county of Dorset for use as asylum accommodation was ended in January 2025 but surplus military sites have been repurposed.

There are fundamental differences between the lives of people seeking asylum living in private sector community-based housing and those living in institutional housing. While both may encounter poor living conditions (24, 25), individuals in hotels and barracks often live in overcrowded conditions where they share rooms or dormitories and toilet facilities with many people (26) rather than self-contained within a room or apartment as was the case for families in community-housing. Those in hotels must eat the food offered, which is provided at minimal cost, at set times (27). They receive around £7 per week ‘pocket-money’ to cover the costs of clothing, toiletries, and transport. Individuals in community housing have access to cooking facilities so can choose what to cook and when to eat, although they receive a meager £49.18 per person per week to cover food, clothing and toiletries. Those living in community housing are more likely to have access to refugee and migrant community organizations (RMCOs), an infrastructure of which has grown up in many asylum dispersal areas across the UK. Barracks are generally located in rural areas away from any forms of support such as RMCOs and healthcare services while hotels are located across the UK in seaside, urban and rural areas, also distant from specialist support mechanisms. Those living in community-based housing should be supported by providers to register with a General Practitioner (GP) upon arrival. While registration can be tricky, once registered they have the same access to healthcare as the general population. Hotel and barracks residents do not have the same level of support and must find their own way to access healthcare or use the Migrant Help helpline which will provide advice but not make connections. Further differences relate to individuals’ ability to make connections to neighbors and access local resources with hotels being so distant from neighborhoods to make connections difficult and some institutional settings subject to attack by right-wing groups (70). Finally, regardless of the form of housing, people seeking asylum are frequently moved around asylum estate, at little notice which can generate feelings of transience (28) and separate them from vital healthcare connections.

## 3 Health, housing and forced migration

Housing and locality conditions are widely acknowledged to be social determinants of health with much research focusing on housing quality or the nature of the neighborhood in which housing is located (29). There is a well-evidenced link between the nature of housing and the health of people seeking asylum (9, 30–35). Physical housing environments facilitate possible behaviors, daily activities, and social interactions and the location of the housing within a neighborhood enables or restricts access to facilities including healthcare resources (36). While having a home can offer a sense of wellbeing, the condition and layout of housing and feelings of safety and belonging also shape ongoing health (35). In a review of scholarship around the connections between health and housing for forced migrants, Ziersch and Due (9) found only eight papers which looked at the relationship between health and housing for people seeking asylum, with none looking at institutional housing. They noted that inappropriate housing facilitated poor mental health outcomes especially where housing was insecure, and individuals lacked social connections. A few studies compare the health outcomes of individuals living in institutional accommodation to those housed within the community. Dudek et al. (37) and Mohsenpour et al. (38) found that institutional accommodation was associated with negative mental health outcomes.

## 4 The hostile environment, structural violence and institutional housing

The hostile environment is a set of policies introduced in 2012 to make living in the UK as unbearable as possible for “illegal immigrants” (39). Despite clear evidence of the devastating effect of the policy on minoritized groups the previous UK Government intensified these policies for people seeking asylum using the unsubstantiated argument that poor conditions will act as a deterrent (40). These approaches include restrictions to the right to work and study, extreme levels of poverty and residence in poor quality housing and can be considered intentionally aimed at those seeking refuge. People seeking asylum have become the main target of hostility politics in the UK with new approaches adopted which simultaneously vilify those arriving and make their everyday lives as difficult as possible. This situation became more marked with the advent of ‘stop the boats’ policy.

Prior to 2018 very few people arrived in the UK by so-called small boats. These are boats which are independently purchased and piloted across the English Channel with most trips organized by so-called people smugglers. With the implementation of increased security measures and a series of penalties for lorry drivers after 2016 it became increasingly difficult for people seeking asylum to cross the channel in the back of a lorry. Between 2018 and 2022 the numbers arriving by small boat increased from 299 per annum to 45,755 (41). In this period at least 64 migrants have drowned (41). Using a somewhat contradictory argument the previous UK Government sought to vilify those who arrive on small boats as ‘illegal immigrants’ coming to take advantage of the UK’s generous welfare system, while stating the need to disrupt the smugglers’ business model to ‘protect’ the vulnerable people who arrive by boat. ‘Stopping the boats’ became their flagship policy in an attempt, some have argued, to scapegoat migrant arrivals and distract from wider structural problems in the UK’s economy and welfare state (42). Successive Home Secretaries called for increasingly more draconian measures to stop the boats and made a connection between the cost of the asylum system and small boat arrivals placing most emphasis on the costs of hotel accommodation. This situation has not changed with the election of a new Government in July 2024.

The use of hotels in the UK exploded during the pandemic. In what initially appeared to be an attempt to reduce dispersal because of the associated COVID-19 health risks, private contractors began to place people seeking asylum in the ready supply of hotels which became available in the absence of tourism. Post COVID-19 this policy continued and by 2023 it was estimated that there were 51,000 people seeking asylum housed in around 400 hotels (43), costing £7 million a day (44) with numbers slightly reduced to 45,700 by the end of 2023 as the Government began to use other forms of institutional housing (3). The expansion in the use of hotels post-pandemic has been linked directly to the increase in small boat arrivals and not the reduction in the use of community-based housing. Indeed, the former Government made claims that people seeking asylum were coming to the UK to live in luxury in hotels with the popular press talking of ‘Channel migrants living in four-and five-star luxury at British taxpayers’ expense’ (45) neglecting to outline the lack of choice in this arrangement or that people seeking asylum are not permitted to work. While some UK charities have drawn attention to the appalling living conditions in hotels and barracks (46) most attention paid to institutional housing concerns the cost.

Hostile policies, including the use of institutional housing not designed for long-term living, and the reclassification of individuals with the right to apply for asylum as ‘illegal immigrants’ alongside the withdrawal of safe and legal routes to asylum in the UK can be viewed as forms of structural violence. Galtung (47) introduced the term “structural violence” to point to structures of inequality, comprising forms of violence, arguing these can also lead to interpersonal violence. The harms occasioned by structural violence are preventable. No individual inflicts the violence, instead the inequality built into structures, herein institutional housing, results in harms. We contend that the use of institutional housing is a form of structural violence in that it generates health harms to individuals residing therein. We outline two forms of structural violence with potential to cause harm to people seeking asylum residing in institutional housing. These are everyday slow violence (40) and violent abandonment (48). We utilize this analytical framework to provide a heuristic with which to make sense of the ways in which the structural violence of policies confining people seeking asylum to institutional accommodation are linked to tangible and preventable harms.

### 4.1 Everyday slow violence

Mayblin’s (40) analysis of the everyday lives of people seeking asylum in UK community-based accommodation showed how asylum support policies operate as forms of slow violence. She demonstrated that the stress of everyday survival on minimal resources was harmful, as individuals were engaged in acts of ongoing survival spending much time seeking resources while depending heavily on their peers to help share the burden. Everyday harms including eating poor food, being contained in the locality, and having poor access to hygiene products were found to be physically and psychologically injurious. Mayblin’s work focused on individuals living in communities who were free to choose their own foods, albeit on a tiny budget, register with a local doctor, and engage with local asylum support. Those residing in institutional accommodation do not have the same, albeit limited, opportunities and must survive off £7 per week. They have no choice of mealtimes or content and restricted access to support networks. We contend that such disempowering everyday experiences, accumulate over time and are likely to cause harm to individuals’ health.

Poor housing conditions have been found to be detrimental to health. Ziersch and Due’s (9) review found that housing conditions such as dampness and overcrowding were common in institutional housing and had detrimental effects on physical health. Bakker et al. (49) found an association between overcrowded institutional housing and poor mental health for people seeking asylum in the Netherlands. Gleeson et al. (50) and Lecerof et al. (51) found that people seeking asylum reporting housing problems had an increased risk of self-reported mental health symptoms. Leiler et al. (52) find that low quality of life in asylum housing was associated with “unpredictable conditions regarding housing location, clearly limited resources regarding healthcare, high levels of passivity and low levels of meaningful daily activities.” (p. 548). Vandevoordt (71) researching the effect of institutional housing conditions in Belgium pointed to the deleterious effect of being unable to choose what and when to eat, or to share food with others. The British Red Cross (5) found people seeking asylum living in barracks felt isolated, with the lack of distraction, and poor facilities reinforcing existing traumas and generating mental health problems. Elsewhere a coalition of refugee and asylum-seeker rights organizations found the most pernicious effect of living in institutional accommodation to be loss of autonomy and agency (53) in terms of choosing where and with who to live, and what and when to eat. Such lack of control alongside enforced confinement is “like prison.” Thus, enforcing conditions which make everyday life difficult can be viewed as a form of slow violence.

There are no routine independent inspections of hotels, with providers required to self-report monthly against a series of performance indicators and quarterly intelligence-led inspections for a small number of hotels (19). Charities working with people seeking asylum in institutional housing in England and Wales have pointed to the health effects of their living conditions. Clinicians visiting barracks reported witnessing a deterioration in people seeking asylum mental and physical health over time. The Chief Inspector of Borders and Immigration found that most residents at Napier Barracks experienced depression and a third had felt suicidal, with people at risk of self-harm placed in decrepit isolation blocks (5). Doctors of the World UK (54) found 74% of residents reported bad or very bad general health diagnoses including musculoskeletal, neurological, respiratory, urological, eye, skin, and digestive conditions. Some 70% of residents had a self-reported psychological condition and a staggering 40% reported suicidal ideation or attempts while in residence. Several were diagnosed with PTSD and reported suffering from flashbacks and nightmares. While the types of institutional housing in which people seeking asylum are forced to live vary markedly, overall evidence suggests that living in such housing generates health harms.

### 4.2 Violent abandonment

A further form of structural violence, termed violent abandonment, refers to inaction in the face of suffering and how it is used as a mechanism of control. Such an abandonment can take place over time and space and in relation to specific situations. Davies et al. (48) show at “the Jungle” camp in Calais, inaction through the intentional withholding of care constituted structural violence. They argue that abandoning migrants in physically and mentally harmful conditions and depriving them of food, medicine, and sanitation, leaves individuals suffering state sanctioned indignities where suffering is normalized. Failure to ensure that people seeking asylum residing in institutional housing have access to the support, including healthcare, they need, may lead to suffering through a form of state-sanctioned abandonment.

People seeking asylum, despite being entitled to the same healthcare rights as ordinary UK residents, are twice as likely to have been denied medical care (23). They are incorrectly denied access due to having no proof of address or because healthcare providers misunderstand their entitlements (23). Researchers point to the UK’s hostile environment role restricting access to healthcare (23, 55). Yet these studies focused on people seeking asylum living within the community, who in theory are supported to access to a General Practitioner. The location of hotels and barracks away from residential neighborhoods may make access more difficult. Little is known about the health and wellbeing of people seeking asylum within institutional housing. The negative impacts of mandatory detention on the mental health of people seeking asylum are well-established (56–59). The extent to which no-choice residence in accommodation described as detention-like has a similar effect has not been clinically assessed.

Given propensity to mental and physical health problems in institutional accommodation, it is particularly important to ensure access to healthcare. Doctors of the World UK (54) found that over 80% of residents in barracks had no access to primary healthcare and 84% did not have the necessary documentation to enable access to free prescriptions. In Penally Barracks, in Wales, the British Red Cross reported that people seeking asylum had no health screening before or after arriving. Residents reported facing long delays to access medical treatment, including those described as being in pain for prolonged periods (5). Elsewhere women were found to have very limited access to maternity care (53). In Australia, MSF found that people seeking asylum residing in temporary institutional-style housing needing to access healthcare, and especially those with mental health problems, did not trust providers who they struggled to differentiate from their ‘captors’.

Between January 2020 and February 2021, British Red Cross teams referenced suicidal ideation or suicide attempts in their case notes for over 400 individuals living in UK institutional housing: almost one person per day (5). They found residents had to ask staff to see a doctor and were expected to disclose their reason for needing medical attention, compromising privacy and confidentiality and leaving them feeling abandoned (5). The Refugee Council (60) staff witnessed people self-harming, in crisis and contemplating suicide. The pressure on mental health services meant accessing support was extremely difficult and individuals were stuck in institutional housing with very limited access to care.

The advent of the COVID-19 pandemic brought a renewed need to think about hygiene and overcrowding. Essex et al. (55) identified the overcrowded living conditions in barracks as representing an extremely high risk of Covid-19, with infected residents unable to self-isolate. During the early stages of the pandemic 33% of Napier Barracks residents interviewed reported having Covid-19 symptoms, of whom 57% were able to access a Covid test with over 50% testing positive. Half of residents reported that there was no way to self-isolate or practice physical distancing because they were housed in rooms with up to 30 people sharing a single toilet and shower (54). People seeking asylum were unable to access to Covid-19 guidance in their own language, while caseworkers lacked knowledge of how to get tests or manage virus outbreaks (54). Whether in the time of Covid, or just in general, it is evident that people seeking asylum in institutional accommodation endure the everyday violence of living in poor and undignified conditions which generates physical and psychological harms around which there is very limited action. Abandoning individuals to conditions known to generate harms and failing to address those harms constitutes structural violence. This is especially important given the continued use of such housing for people seeking asylum.

## 5 Materials and methods

As stated earlier, our overall aim is to explore health and access to healthcare for people seeking asylum in institutional housing, situating this analysis within the frame of structural violence. To achieve this, we analyzed secondary quantitative and qualitative data collected by Doctors of the World UK (DOTW UK), a medical charity that aims to provide immediate healthcare support while connecting those excluded from healthcare into NHS services, to ensure their physical and mental well-being. For this paper, we use data that DOTW UK have collected about a specific subsection of the people they seek to support people in institutional housing. Our mixed methods approach to data analysis uses the quantitative data collected from questionnaires and, unique to this study, a medical examination, to provide a descriptive picture of DOTW UK service users, as well as outlining aspects of structural violence perceived as effecting health and healthcare access, and qualitative data to provide more depth to our findings, richer descriptions. In the sections below, we delve further into our approach.

### 5.1 Data

Here we briefly describe the data that we used, but we invite you to consult (72, 73) for a further detailed description. The paper is based on a post-hoc analysis of data collected by DOTW UK, following an invitation from them to use this data to develop a greater understanding of health status and access to healthcare in institutional housing. The data consists of routine data collected by DOTW UK between July 2020 and January 2022 when seeking to address the healthcare needs of people seeking asylum living in institutional accommodation by providing services and medical assessments. GP and case work volunteers and staff members collected data from individuals calling DOTW UK’s advice line, from remote consultations with individuals residing in barracks, and face-to-face consultations at two hotels, all in England. We refer to these individuals as service users (SUs) throughout. Information was analyzed for the SUs who gave consent for data usage.

Demographic, social and medical quantitative information were collected using DOTW UK standard questionnaires. These questionnaires utilize standardized health questions also employed in the UK’s Census and questions that the Médecins du Monde international network developed themselves which are specific to the health of migrant populations and which they have used since 2006 (74). To the best of our knowledge no validated instruments of this nature exist, thus DOTW developed their own. The DOTW UK questionnaires, which are used for documenting all of their consultations, comprise of an ‘administrative’ questionnaire, where information about the consultation and demographic information about SUs is recorded, a ‘social’ questionnaire, where information about the living conditions (including health status), activities, immigration situation, and healthcare access of SUs is collected, as well as a ‘medical’ questionnaire, which is completed for SUs requiring a medical consultation as part of their appointment and includes a variety of health-related questions. Previous research on the health status of people seeking asylum has relied on self-rated health measures which are inherently subjective with questions raised about their validity, especially for minority ethnic populations (61). All staff and volunteers doing the medical consultation were qualified doctors or nurses. An additional questionnaire was completed for every SU residing in one of the hotels (‘hotels questionnaire’), which covered further information about health and healthcare. Questions asked can be found in Supplementary Table A1.

We also used qualitative data in the form of information about the consultation (e.g., contextual information, actions undertaken) written by the DOTW UK staff members or volunteers in the form of free-text notes. Note that data comprises the volunteer’s record of the interaction, which can be understood as a subjective account of the consultation, even when using questionnaire instruments, and could lead to information bias. Nonetheless the data we analyzed is comprehensive, providing information based on medical examination and social situation.

We analyzed data for 313 SUs living in institutional housing having undergone a consultation with DOTW UK who consented for their data to be used for research.^1^ This included health-specific information of 85 SUs for whom the medical questionnaire was completed following an examination, as well as the additional information provided by the hotels questionnaire for 257 SUs in hotel accommodation. Free-text notes from a sample comprising about a third of SUs (*N* = 106, 82 in hotels, 10 in barracks, and 14 advice line) and ranging from 1 to 30 pages in length were also analyzed. Each set of notes contained a record of all contacts with the SU relating to their attempts to address the SUs problem.

This data is, to the best of our knowledge, the only data of this kind collected to assess the actual health and wellbeing of people seeking asylum in institutional asylum accommodation at the time of writing. It details medical conditions, offers an account of the conditions faced by respondents and the actions taken by DOTW UK to address medical conditions although it does have significant limitations discussed in section 7.2. Nonetheless the insight it offers into medical and social conditions can be used to shape further research.

### 5.2 Analysis

The quantitative data was analyzed through appropriate descriptive statistics (distribution and frequencies for univariate and bivariate statistics given the categorical nature of the variables), with results based on small cell counts (N ≤ 5) excluded. We adopted a systematic thematic approach to qualitative data analysis whereby we read 10% of the free notes, devised codes and then applied these to the existing data plus a further 23%. We added in new codes as necessary and agreed not to analyze further notes having reached saturation (see Supplementary Table A.2 for coding frame).

### 5.3 Ethical considerations

Ethical approval was received from DOTW’s own ethics procedures and that of the University of Birmingham. As mentioned above, results from the quantitative analysis avoided reporting small cell counts to avoid identification of SUs in the results. For reasons of confidentiality, we are not permitted to include verbatim quotations or share case studies with identifiable details because such consent was not requested when DOTW UK originally collected the data. The latter would have been informative, revealing the extent to which individuals with complex health conditions struggled to manage those conditions, some of which were life threatening. Nonetheless, the combination of survey data and the volunteer’s notes are unique and bring new insight.

## 6 Results

We consider results from all strands of the work, with quantitative data providing a macro-level, descriptive, overview, and qualitative providing more detailed experiences. We present our findings through the lens of structural violence, more specifically slow violence and violent abandonment. Table 1 provides brief details of the operationalization of our results using this lens, with more detailed information found in Supplementary materials. We group questions about, and mentions of, health status, mental health needs and mentions of living conditions as aspects of slow violence. We consider violent abandonment through various themes such as reasons for consultation, the presence of chronic illness, barriers to healthcare, any resolution of outstanding issues, as well as knowledge about healthcare (or lack thereof) and isolation. We first, however, provide further information about the characteristics of the SUs whose data we analyzed, before delving deeper into slow violence and violent abandonment.

**Table 1:** Mapping of indicators and themes to analysis components.

Analysis components	Indicators/themes
Characteristics of SUs	Immigration status
	Location of data collection
	Sex
	Age groups
	Length of time in UK
Slow violence	General Health
	Mental health needs
Violent abandonment	Support sought
	GP registration
	Chronic illness
	Urgent illness
	Barriers to healthcare
	Information about Covid-19
	Knowledge re: where to see prescriptions
	Help with health costs
	Source of information

Further details about this mapping can be found in Supplementary materials.

### 6.1 Characteristics of service users in the data

Characteristics of the SUs are represented in Table 2. The sample of SUs comprised mostly individuals classified as non-EU people seeking asylum (just under 95%). A large share (82.1%) of the SUs in the data had attended a face-to-face consultation in a hotel, whereas smaller shares received a consultation through the advice line (9.6%) or while residing in barracks (8.3%). Most SUs needing consultation were men (about 75%) and young (56% under the age of 30). Only a third of SUs had been in the United Kingdom for under 2 months, with some (21.1%) having been in the UK for several months—or even years.

**Table 2:** Characteristics of service users in the sample.

Immigration status (*n* = 249)	% distribution
Non-EU person seeking asylum	94.8
Other	5.2
Sex (*n* = 303)	% distribution
Female	25.4%
Male	74.6%
Location (*n* = 313)	% distribution
Advice line	9.6
Barracks	8.3
Hotel	82.1
Time spent in UK (*N* = 299)	% distribution
Under 2 months	33.4
2–3 months	28.4
4–5 months	17.1
6 months +	21.1
Age groups (*N* = 313)	% distribution
0–17	8
18–24	23
25–29	25.6
30–34	16.3
35–39	10.2
40–44	8.3
45–49	4.5
50+	4.2

### 6.2 Slow violence

In terms of slow violence, we outline the daily harms that accumulate from stress and lack of resources, with a specific focus on food. We also examine health status and health issues highlighted during the consultations.

Just over half of the SUs were reported as having good or very good health, with the remainder reported as having fair or bad/very bad health. The evaluation of the general health of the service users is quite different from the general population (62), with a lower share having good or very good health (50.2%/74.6%) and a higher share having bad or very bad health (27.1%/7.4%). Whereas this cannot be a direct comparison, it could suggest that SUs appear to be likely to have lower perceived general health.

Among people residing in hotels, close to 32% were reported as having a mental health need that needed to be addressed. For context we note this was slightly higher than the rates reported in the general population at around 27% (63). Mental health conditions were referred to in 56 entries, with common issues including anxiety, PTSD, depression and sleep disruption. Suicidal ideation, self-harm, and feelings of depression were reported, with the latter leading to lack of appetite. In many instances mental health conditions were said to have either arisen or been exacerbated by living in the accommodation.

#### 6.2.1 Health and quality of accommodation

Mentions of the quality of accommodation, including the food provided, and its impact on SUs, were often found in the notes. The food was described as being of low quality and very different from the SUs’ usual diet. We heard that the food was not fresh, lacking in fruit and vegetables and extremely unpalatable. It was also provided without any flexibility with mealtimes determined by providers and no food available at other times. Those who missed meals because of immigration, health or other appointments go hungry. Food was linked to a wide range of problems including: weight loss, stomach pain and rectal bleeding. The provision of diet-specific food, even for medical reasons, required permission from the Home Office, which determined the food on offer. In one case, permission for medically required liquid food took over a week to arrive. The notes describe that particular SU experiencing weight loss and weakness while waiting for permission. In another case, long-term food refusal among a young SU, had a detrimental effect on the child’s condition, but was not addressed. Some SUs struggled without access to cash, unable to purchase basic clothing or toiletries or buy any food despite finding the food provided inedible. The following excerpt from the volunteers’ notes offers an example of the kinds of issues that residents faced around food:

The food they receive is bad. SU said that she does not think they cook food there, they just receive frozen food that they heat up. SU received very bad allergy after a meal there and she was very bad that they even had to call an ambulance. Ever since then she avoids to eat the food here and just drinks tea and coffee. I asked the SU to speak with the hotel manager and explain that they have health problems with their bowel and that they need to be on a special healthy diet. SU said that she has spoken with everyone and they do not care.

The notes covered how SUs referred to the ways in which living conditions were seen as undermining their mental and physical health. Overcrowded housing conditions, especially in barracks where up to 25 people in a room would share two toilets, were recorded in the volunteer’s notes ‘2 toilets/25 pp.; do have access to masks; poor ventilation’. This was especially linked to concerns about COVID-19 and self-isolation. Fear of living in hotel and barrack accommodation was reported by several SUs who experienced the feeling of being re-imprisoned, contributing to a decline in their mental health. Loneliness and feelings of isolation were also noted as exacerbating mental health conditions. Physical health concerns linked to the accommodation were also mentioned in the notes, including the appearances of rashes as well as respiratory problems associated or exacerbated by living conditions. In addition, women living in mixed gender hotels did not feel safe with no special measures made to ensure their protection from harassment, a situation shown elsewhere to result in high levels of stress (64). In the volunteer notes below we see an account of a woman who does not feel safe to leave her room even for meals. This situation and the associated isolation and lack of sustenance was influencing her mental and physical health:

SU says that she has been followed by a man in the hotel and does not feel safe. Just stays in her room and goes down to reception to get milk.

As we can see, the everyday living conditions in hotel and barrack accommodation were highlighted in grim terms, especially regarding the way in which some of these elements were linked to health in the notes. The experience of these everyday harms, and their compounding effects over time, were reported to harm the health of SUs.

### 6.3 Violent abandonment

Regarding violent abandonment, we explore the ways in which SUs faced inaction in meeting their health needs.

#### 6.3.1 Lack of support

Help with GP registration was one the most often mentioned reasons for consultation with DOTW UK in the analysis of the social questionnaire, not surprising given the low share of SUs registered with a GP. Other needs included assistance with completing documentation for help with health costs and help to access other health-related services such as a dentist, counseling, or an optometrist. For SUs requiring a medical consultation (85 service users), 22 were diagnosed with at least one chronic condition and 23 with an urgent one. Mental health conditions were the most often mentioned for medical consultation.

The sample of notes analyzed confirmed the picture outlined above, allowing more in-depth information. The needs for engaging with the DOTW UK service ranged from accessing prescription medicine or sanitary products; needing medical letters confirming the unsuitability of hotel accommodation; pregnancy and access to antenatal care; experiencing domestic violence; requiring an interpreter for medical care; needing medical care for a child or other relative; and needing to arrange a COVID-19 test or vaccination. Concerns about health conditions, including skin problems, allergic reactions, hypertension, diabetes, Hepatitis B, headaches, migraines, epilepsy, congestion, and appendicitis, were also mentioned. The absence of support to access healthcare and social care provision from accommodation providers meant that DOTW UK had to work to connect SUs to the services they needed. The notes below show how one individual was abandoned by the hospital and the hotel leaving her without help for her extensive and complex healthcare needs:

She has been discharged back to the hotel as the hospital are saying they cannot treat her as she ‘does not have an address’ and is not registered with a GP. The hotel are refusing to provide proof of address and the GP will not register her.

The analysis of the notes confirms the extent and complex nature of SUs’ needs. Many SUs required support for more than one reason. Contacts could involve a simple request or be extremely complex with individuals having multiple conditions, symptoms and needs that demanded a complex range of interventions. The heavily anonymized example given below from the volunteers’ notes details the range of problems faced by one individual:

Has been in the hotel for anonymized months. Few medical issues they need help with:

1. Anonymized problem with anonymized eye causing impaired vision, never had assessment or treatment for this.
2. Stomach aches, worsened on coming to the UK and concerned the water is causing it.
3. Previously broken anonymized and had surgery in anonymized in Anonymized. Occasionally getting some pain and also getting some pain in anonymized as well.
4. Anonymized not broken, not causing pain but (discomfort).

The nature of the consultation could involve a multitude of health conditions needing care, as well as access to many different prescription medications, for a SU without access to medication. While DOTW UK was able to arrange for prescriptions, the hotel would not allow controlled or refrigerated medication leaving individuals with no route to access life sustaining drugs.

#### 6.3.2 Barriers to healthcare access

The need for a DOTW UK consultation often implied an inability to access healthcare, quite often due to being in hotel or barrack accommodation as people were not receiving healthcare on site to meet their needs and struggled to access local GPs. Some notes indicated that even when in considerable pain residents were ignored. This was observed in the volunteer’s notes ‘Had appendicitis in November, he was in lots of pain, but he says the [anonymized] staff ignored him’. The questionnaire data showed that barriers to accessing care most often mentioned were lack of knowledge of the healthcare system, language barriers, and administrative barriers. While the first two concerns are likely to affect people seeking asylum in community housing, administrative barriers are particularly problematic for individuals living in institutional housing because they lack a proof of address. Language was often mentioned as limiting access by SUs not only to healthcare, but to health-related information. In the hotel questionnaire data, 52.5% of people residing in hotels did not have access to information about COVID-19 in their own language.

Caseworker notes showed that issues with healthcare access continued to be experienced despite GP registration, even for those receiving hospital care. The notes indicated that those with GPs did not know the address of the surgery or how to get there or how to make an appointment, for example ‘SU does not know what GP he is reg with and how to contact them … Has not spoken to GP yet, remained confused about how to book an appointment’. Information was lacking about services or the outcome of medical consultations, especially in an appropriate language as illustrated by the following example from the volunteer’s notes ‘she has not managed to book apt (sic) with GP because of language barrier (she called asking for interpreter and they did not do anything)’. Knowledge about the functioning of healthcare systems, including where to seek support and the ability to use online forms available only in English, was also raised.

Knowledge about accessing healthcare and health-related services was important, regardless of GP registration status. Some 83.1% of SUs residing in hotels did not know where to get a prescription for an existing health concern and only 26.7% of people had the necessary certificate to help with health costs. The volunteer notes gave examples of SUs who were given prescriptions but could not afford to pay for them, unaware of their free medication entitlements.

Given the apparent need for knowledge, SUs residing in hotels were asked who they would go to for healthcare advice. Among the answers provided as source of advice, 70% mentioned the hotel staff, 13.2% said they did not know, 7.8% would ask other residents, 6.6% would look online, and 4.3% would ask friends.

Life can be threatened by failure to provide timely access to healthcare and medication. The lack of helpfulness on behalf of hotel staff as well as their mistrust regarding the reporting of medical conditions were mentioned several times. In the instance detailed below from the volunteer’s notes, DOTW determined that a SU needed access to emergency care and the hotel refused to assist.

Explained concerns and stressed the importance for SU to attend A&E today. Manager says that cannot arrange a taxi as Home Office needs a 48 hours notice.

In some instances, professionals such as health visitors and midwives were reluctant to visit patients at the hotels, despite requests from DOTW UK.

#### 6.3.3 Responding to abandonment

Within all the accommodation sites DOTW UK was the only route to advice about how to access healthcare. Only very limited healthcare in barracks and no healthcare in hotels was provided beyond the DOTW UK consultation. DOTW UK acted as a mediator, attempting to facilitate residents’ access to healthcare. They undertook a wide range of actions to try to address needs. These varied from resolving a health problem through securing access to services or medication, to trying to resolve the underlying structural factors generating health problems, such as diet or being re-traumatized by living in detention-like accommodation. They made appointments with GPs and followed up appointments with primary or secondary care. They booked COVID-19 tests and vaccines, helped to access medication, offered STI/HIV testing, and arranged interpreters. They tried to arrange safeguarding for vulnerable residents, wrote letters on their behalf, helped with online referrals, and tried to help children access schooling or appropriate accommodation. The accommodation providers, hotel workers, the Home Office or their contracted agents were said to make little effort to support residents to access these or any other services, essentially abandoning them.

Living within institutional accommodation, people seeking asylum did not have access to a caseworker, so DOTW UK engaged in large amounts of casework focused on securing healthcare access and improving the residents’ wellbeing, much of which went well beyond their usual remit. Of the 106 cases reviewed in the qualitative analysis, on 20 occasions DOTW UK took just one action on the part of a resident, but often complex problems demanded several actions. In 62 cases 2–5 actions were needed and in 21, 6–10. Each action might comprise multiple telephone calls, referral letters and e-mails and thus engagement would continue over several months. Resolution was achieved within one interaction for 55 service users, for 18 it was achieved over a month and for 28 service users it took between 1 and 6 months. One very complex case took over 6 months to resolve. Ninety out of 104 cases were resolved, 5 were not and in 7 instances DOTW UK lost contact with the SU. On 36 occasions, the work involved referral to other organizations with the volunteer using a combination of telephone and e-mail to secure referrals. DOTW UK worked extensively with local charities. These services often did not respond to a simple referral because they were operating over capacity following the opening of several asylum hotels in their locality. DOTW UK also connected with schools, health visitors, social care, multiple functions within the National Health Service, the Home Office, solicitors, sexual health clinics, emergency services, consultants, and psychiatrists. They referred to Modern Slavery services and received several referrals from the Human Rights Network. DOTW UK engaged in all of the above work because individuals living in institutional accommodation were abandoned, in geographical areas without an infrastructure familiar with the specific needs of people seeking asylum. In some instances, where DOTW UK linked people with urgent healthcare or helped them to access life-preserving medication, we might argue that the violent abandonment within hotels had the potential to result in further harm or even death, of individual residents.

## 7 Discussion

### 7.1 Overview and analysis

To the best of our knowledge our analysis is the first to apply a structural violence lens to the analysis of the health of people seeking asylum resident in institutional housing and the first to utilize qualitative and quantitative information collected by a medical doctor and comprising of medical consultations. Our findings outline the health issues and barriers to healthcare access of people seeking asylum residing in institutional housing, even the hotels widely portrayed as a luxury and “a pull factor.” Housing is a key determinant of health (9), and in the case of institutional housing, slow violence including overcrowding, poor food, absence of choice, isolation and lack of distraction is likely to be linked to both poor physical and mental health outcomes. Institutional housing provided little more than a roof over the heads of people seeking asylum. We contend that the nature of conditions in this housing and the lack of systematized access to healthcare are health harms. The treatment of individuals in such housing is thus framed as a form of structural violence wherein the state supports only mere life and in its failure to provide decent conditions and access to healthcare generates interpersonal harm.

In some instances, conditions were so bad, that individuals attempted to take their own lives or spoke of their desire to do so. In other situations, life and long-term health were reported as threatened by the abandonment of sick and vulnerable individuals via the failure to provide timely access to healthcare. In some cases, denial of special diets or vital medications were linked to suffering. The abandonment of individuals to fend for themselves to access healthcare, source medication, access schooling, antenatal care, and other services, is a form of structural violence, which despite DOTW UK’s efforts to intervene were reported as resulting in further physical and mental harms. The failure to act in the face of suicidality and self-harm when psychological health problems are generated or exacerbated by everyday slow violence and require multiple interventions from DOTW UK as navigator, constitute violent abandonment (48).

While access to housing is a human right for all (OHCHR, undated), those living in institutional accommodation were denied key aspects of those rights in the form of privacy or opportunities for development and social integration (7). Institutional accommodation operated as a barrier to accessing healthcare because those placed in such housing were not given access to mechanisms by which they could access healthcare. Green et al. (65) have demonstrated that even native residents require navigational support to understand complex healthcare systems. Elsewhere Shim (66) talks of the cultural health capital needed to enable meaningful access. Within institutional housing, people seeking asylum, many with complex and urgent healthcare needs, and unable to communicate in English, were abandoned to their own devices to understand the system, identify, and register with a GP and argue for their rights to free care. These attempts occurred without being offered any support to access the documentation they were told they needed, and which could have been made available by the hotels in which they resided. In the absence of systems within asylum support to facilitate access, DOTW UK acted as a navigator often dealing with health problems requiring multiple interventions over several weeks. DOTW UK are a voluntary organization with limited capacity and were able to provide services to SUs in hotels in two London boroughs for a fixed period. It is impossible to know how the 10,000 s of individuals currently residing in institutional housing access urgent care or life-sustaining medications without such interventions.

The UK Government recognizes the importance of safe and secure housing as an indicator of refugee integration (8). Yet they maintain a position that integration can only begin when an individual gains refugee status (8). They refuse to engage in discussion about the harms occasioned to people seeking asylum, despite existing evidence of the long-term effects of structural violence for those in community-based housing (49). Given that many of the people seeking asylum currently residing in institutional housing will gain some form of refugee status as their presence in the UK predates recent legislation that denies most people access to the asylum system, it is important to highlight the damage that slow violence and abandonment seem to be occasioning to individuals who eventually will be expected to integrate. The untreated psychological harms, re-traumatization, and deterioration in physical conditions (i.e., irreversible damage to eyesight without appropriate diabetic care) will most likely inevitably undermine long-term integration prospects and quality of life. Rather than the UK Government expanding the use of institutional housing and making it more basic as per the current strategy, there are moral, ethical and socio-economic rationales for limiting its use. Where institutional housing must be utilized, it must be more humane and facilitate access to healthcare and basic resources such as decent food (67).

The right to health applies to everyone, regardless of immigration status, and is well established in international treaties and standards including access to health services and wider areas that impact upon health (67). The UK government has international obligations to protect the right to health under the Universal Declaration of Human Rights and the UN International Covenant on Economic, Social and Cultural Rights (ICESCR) and the European Convention on Human Rights (68). The UN Committee on Economic, Social and Cultural Rights describes the right to health and set out examples of how the right to health should be upheld. These include ensuring that health facilities, goods and services are accessible to everyone without discrimination, within safe physical reach for all sections of the population, especially vulnerable or marginalized groups and making sure that health services are culturally appropriate, and that healthcare staff are trained to recognize and respond to the specific needs of vulnerable or marginalized groups. They also suggest the State meets its obligations in sharing appropriate information relating to health issues, healthy lifestyles and nutrition, the availability of services and supporting people in making informed choices about their health (69).

It is important to highlight the erosion of these rights under conditions of hostility and with neo-liberal attempts to cut costs and their consequences for public health. The consequences of not facilitating these rights are state-sanctioned abuse of rights for a category of persons classified as vulnerable and for whom the UK Government has international obligations. Such treatment sets a dangerous precedent. If rights can be removed for one group, they can be removed from others. The core principles of public health, especially around protection of the vulnerable and prevention of disease and death cannot be realized within current approaches to healthcare provision and access in institutional housing. From a moral perspective we have a duty to do no harm yet individuals residing in institutional housing experienced multiple harms. From a socio-economic perspective the health harms linked to institutional housing may have lifelong effects if individuals experience irreversible effects, for example, from struggling to access diabetic or psychiatric medications. Effects may leave individuals permanently disabled and prevent them from engaging in volunteering, work, and language learning, which the Government consider the main means of becoming integrated into life in the UK once they have gained status (8), as the majority of people whose asylum claims were processed did in 2023^2^ (2). Further from an entirely pragmatic perspective, long-term mental and psychological conditions have treatment cost implications, which could be reduced or eliminated with swift and humane access to healthcare.

### 7.2 Recommendations

Decent quality housing and systematized access to healthcare for all should be the Government’s policy aim. People seeking asylum should be accommodated in a humane way that enables meaningful access to full NHS care services to meet health needs and provide continuity of care. The Home Office should introduce a centrally funded system that houses people seeking asylum in safe and sanitary housing in communities across the country where they can access decent food and toiletries and that enables access to local GP and specialist health services.

There are ways that current mechanisms can improve conditions for people seeking asylum in institutional housing.

1. Faster, but fair, assessments of asylum claims would enable people to move on from institutional housing more quickly.
2. Automatically enrolling people seeking asylum with GPs or amending Home Office contracts with accommodation providers to include provision of direct support for GP registration would enhance access.
3. Locating NHS healthcare professionals within institutional housing would enable better access to healthcare.
4. Automatically providing health costs certificates for a minimum of 12 months would enable people to immediately access vital medications.
5. Obliging hotels to provide facilities for restricted and refrigerated medications would prevent the denial of life-sustaining drugs.
6. Making hotels responsible for providing medical diets within 48 hours of prescription and without asking for Home Office permission would support the health of those currently unable to access food.
7. Improving the quality of food, the flexibility of mealtimes and enabling open access to healthy food would enable people to have a little more agency and to eat more healthily.
8. Providing resources to enable distractions by funding local charities to offer befriending and activities, such as those offered by Napier Friends and Care4Calais, would provide relief from isolation and boredom.
9. Ensuring those with mental health conditions are not housed in institutional housing may reduce levels of self-harm and suicidality.

All these suggestions would require the new Government to adopt a more humanitarian approach to the protection of people seeking asylum.

### 7.3 Limitations

Our study utilized unique data that to the best of our knowledge is not available elsewhere. However, there are several limitations. The study was a post-hoc analysis of data collected and thus we were unable to develop an a-priori research plan. The questionnaires used are included in Supplementary materials, but they are not fully standardized or validated and thus direct comparison with other studies or the general population is not possible. The study is hard to replicate given the reliance on access to people seeking asylum and the ability to engage in medical examinations.

The indirect mode of reporting, the relatively small number of responses, and the rather limited locations from which the data was collected (e.g., housing locations, geographic location) limit the extent to which we can generalize from our findings. Information reporting and response bias may be a problem given they were made by volunteers and thus filtered through their perspective. For reasons of confidentiality the data are not openly available, which makes reproduction/reanalysis difficult. A further limitation is the lack of systematic consideration of important factors such as religion, ethnicity, language, prior torture, and time of stay and wait for an asylum decision, pre-migration mental health prior, and family status (alone, with family members) which we were unable to access as this data was not collected. Finally, the data only covers those who approached DOTW UK for help excluding individuals unable to locate DOTW UK, or residing in a hotel where they do not operate. Our analysis excludes those who do not have a need for DOTW UK’s services.

### 7.4 Future research

Our work might be characterized as a pilot given its small scale post-hoc nature. Results should be seen as a guideline for further systematic research, both qualitative and quantitative. Further work in collaboration with DOTW UK would look to validate and standardize the instruments used ensuring that they can be compared with the general population and other relevant studies, and also to gain ethical approval to use verbatim and detailed case accounts. Research is needed to engage directly with a wider population of people living in a wider range of institutional housing and on a longitudinal basis to examine the long-term consequences of structural violence. Further research could also compare the health of those living in community-based housing to those in institutional housing. It is worth noting that at the current time access to all individuals living in Government-provided housing is highly restricted.

## Data Availability

The data analyzed in this study is subject to the following licenses/restrictions: data belongs to DoTW UK and cannot be shared beyond the research team.

## Footnotes

^1^Note that, due to missing information, the total sample size in the quantitative results varies from one question to the next and no imputation methods were used.

^2^Over 70% of asylum claims in the UK were successful in 2023 (2).
