# Supplementary material for: “They just left me”: people seeking asylum, mental and physical health, and structural violence in the UK’s institutional accommodation
Source: Front Public Health. 2025 Mar 12;13:1454548. doi: 10.3389/fpubh.2025.1454548 (PMC11936787; doi:10.3389/fpubh.2025.1454548)
Supplement: Supplementary file 1 [file Table_1.DOCX]

Supplementary Material

# Supplementary Tables

# Table A.1 Questions in the DOTW UK Data

| **Administrative information (present in social & medical forms)** |
| --- |
| User id |
| Date of consultation (+ first year of consultation) |
| Sex |
| Date of birth |
| Age |
| Use of interpreter |
| Language of interpreter |
| Borough of residence |
| **Social questionnaire** |
| *Living conditions* |
| Type of accommodation |
| Children: have any |
| Children: live with person |
| Children: how many |
| Children: where live if not with person |
| General health |
| Little interest or pleasure in doing things |
| Feeling down, depressed or hopeless |
| *Activities and resources* |
| Average amount of money to live in past 3 months above poverty |
| *Administrative/Immigration situation* |
| Nationality (+ country) |
| Length of residence (days & years) |
| Immigration status |
| *Health cover and obstacles to accessing healthcare* |
| Chargeable healthcare costs |
| Problems in accessing healthcare and healthcare services: last 12 months |
| Information about contraception/family planning? |
| Support provided (type + further details) |
| When did you last see a dentist in the UK? |
| When did you last have an eye test in the UK? |
| Would you like screening today for HIV and STIs? |
| Would you like to be invited back for a chest x-ray? |
| **Medical questionnaire** |
| *Medical history* |
| Want information on contraception (+ details) |
| Would like contraception (+ details) |
| Pregnancy status (+ termination info from 2012-2015) |
| Pregnancy: weeks |
| Pregnancy: access to antenatal care (+ reasons if not) |
| Experience of FGM (+ when/where) |
| *Preventative questions* |
| Tests: Hep C (including results + date) |
| Tests: Hep b (including results + date) |
| Tests: HIV (including results + date) |
| Tests: Tuberculosis (including results + date) |
| Date of the last smear test |
| Experiences of violence (detail + when occurred + further details) |
| Health problem/result of consultation: diagnosis required |
| Diagnoses: in words |
| Diagnoses: ICPC code |
| Have been vaccinated against Tetanus |
| Have been vaccinated against Hepatitis B |
| Have been vaccinated against Measles, Mumps and Rubella |
| Have been vaccinated against whooping cough |
| Have been vaccinated against Polio |
| If yes, are there any children currently at risk? |
| if yes, and a migrant, was this violence one of the reasons you came to the UK? |
| what actions have been taken today? |
| **Hotels questionnaire** |
| How long have you lived in the UK |
| Are you currently registered with a GP? |
| Have you had any healthcare since arriving in the UK? |
| Where do you go if you or your family member feel unwell? |
| How many times have you moved since you claimed asylum? |
| Where do you get information about accessing healthcare? |
| If you or a family member has needed hospital treatment since you claimed asylum can you describe the process? |
| Do you take medication? |
| Do you know how to get your next prescription? |
| Do you have HC2 cert? |
| Do you receive S95 support? |
| Have you accessed Covid-19 support in your language? |
| Do you identify as having a mental health need? |

# Source: Lessard-Phillips et al. (2022)

Table A.2: Coding frame for volunteer notes

| Health conditions |
| --- |
| Gender and pregnancy |
| Hotel or Barracks |
| Reasons for contact |
| Actions taken by DOTW |
| Resolution or outstanding issues |
| Barriers |
| Living conditions (including food, safety, access to hygiene etc.) |
| Other organizations involved |
| Time to resolve |
| Number of interventions |
| Other |
